# Indole-3-carbinol alleviates allergic skin inflammation via periostin/thymic stromal lymphopoietin suppression in atopic dermatitis

**DOI:** 10.1186/s13020-024-01042-5

**Published:** 2024-12-26

**Authors:** Yun-Mi Kang, Hye-Min Kim, Junho Lee, Jong-Suep Baek, Minho Lee, Hyo-Jin An

**Affiliations:** 1 Department of Oriental Pharmaceutical Science, College of Pharmacy, Kyung Hee University, Seoul, 02447 Republic of Korea; 2 Department of Herbology, College of Korean Medicine, Sangji University, Wonju, Gangwon-Do 26339 Republic of Korea; 3 Korean Medicine (KM)-Application Center, Korea Institute of Oriental Medicine (KIOM), 70 Cheomdan-Ro, Dong-Gu, Daegu, 41062 Republic of Korea; 4 Department of Bio-Health Convergence, Kangwon National University, Chuncheon, 24341 Republic of Korea; 5 Department of Life Science, Dongguk University-Seoul, Ilsandong-Gu, Goyang-Si, 10326 Gyeonggi-do Republic of Korea; 6 Department of Integrated Drug Development and Natural Products, Graduate School, Kyung Hee University, Seoul, 02447 Republic of Korea

**Keywords:** Atopic dermatitis, Indole-3-carbinol, TSLP, Periostin, DNCB, Keratinocyte

## Abstract

**Background:**

Atopic dermatitis (AD) is a chronic multifactorial inflammatory skin disorder with a complex etiology. Despite its increasing prevalence, treatment of AD is still limited. Indole-3-carbinol (I3C) is found in cruciferous vegetables and is formed when these vegetables are cut, chewed, or cooked; it exerts diverse pharmacological activities.

**Methods:**

HaCaT keratinocytes stimulated with tumor necrosis factor-α and interferon-γ mixture and NC/Nga mice stimulated with 2,4-dinitrochlorobenzen (DNCB) were used for AD models, in vitro and in vivo, respectively.

**Results:**

The results showed that I3C reduced the expression of pro-inflammatory cytokines, thymic stromal lymphopoietin (TSLP), and periostin in in vitro model. Oral administration of I3C alleviated AD-like skin inflammatory symptoms, including serum IgE levels, epidermal thickening, inflammatory cell infiltration, transepidermal water loss, and scratching behavior. Moreover, I3C decreased the expression of TSLP and periostin and recovered the expression of skin barrier proteins by regulating Aryl Hydrocarbon Receptor and inhibiting the mitogen-activated protein kinase and nuclear factor-κB pathways in the skin of DNCB-induced AD mice.

**Conclusions:**

I3C is suggested as a potential therapeutic alternative for the treatment of AD by repressing allergic inflammatory pathways.

**Supplementary Information:**

The online version contains supplementary material available at 10.1186/s13020-024-01042-5.

## Background

Atopic dermatitis (AD) is a long-standing and periodically recurring inflammatory disorder of the skin, which has seen a notable rise in incidence over recent years [1]. The pathogenesis of AD involves both impairment of the skin barrier and allergic inflammation of the skin, with the T-helper (Th) 2-type immune response playing a predominant role in the dermatological manifestations of the condition [2]. A wide array of allergens have been implicated in triggering AD, encompassing dietary allergens, airborne allergens, infectious agents, and physical irritants [3]. The immune response to these allergens involves both the innate and adaptive immune systems. It is widely acknowledged that repeated or continuous exposure to these allergens can lead to persistent chronic allergic inflammatory conditions [4].

Formerly regarded solely as constituents of the skin's protective barrier, keratinocytes are now recognized as integral components of the innate immune system, actively engaging in the inflammatory processes of AD by secreting pro-inflammatory cytokines and chemokines [5]. Notably, cytokines produced by activated keratinocytes, especially thymic stromal lymphopoietin (TSLP), are pivotal in triggering or enhancing Th2 immune responses, as well as driving the inflammatory processes in AD by mediating interactions between keratinocytes and dendritic cells (DCs) [6]. TSLP, akin to interleukin-7 (IL-7), is synthesized by epithelial cells residing at the barrier interfaces of the skin, lungs, and gastrointestinal tract [7] emerging as a key regulator of AD, notably abundant in keratinocytes within AD-afflicted human skin [8]. Furthermore, the induction of AD-like dermatitis in TSLP transgenic mice underscores its significance [9], while the absence of TSLP receptors in mice leads to a failure in developing allergic skin inflammation following epicutaneous sensitization to allergens [10]. Additionally, periostin, an extracellular matrix protein belonging to the fasciclin family, exhibits heightened expression in the skin of AD patients, with its levels significantly correlated to disease severity. It plays a crucial role in sustaining and exacerbating allergic skin inflammation by facilitating the production of TSLP and promoting Th2-driven immune reactions [11]. Periostin not only stimulates keratinocyte proliferation and viability but also directly triggers TSLP production [12], thereby creating a feedback loop that reinforces the Th2 immune response and keratinocyte activation within the AD pathology.

Indole-3-carbinol (I3C) is abundant in cruciferous vegetables; broccoli, brussels sprouts, cabbage, collards, cauliflower, kale, mustard greens, turnips, and rutabagas. I3C is formed when these vegetables are cut, crushed, or cooked by the breakdown of glucosinolate glucobrassicin [13]. Within the gastrointestinal tract, I3C undergoes conversion into a biologically active dimer known as 3,3′-diindolylmethane (DIM) when ingested. Several studies have indicated that I3C has therapeutic potential for both the prevention and treatment of cancer as a chemopreventive agent in preclinical cancer models [14]. I3C and its metabolic derivatives suppress the proliferation of various cancer cell lines by targeting a wide spectrum of signaling pathways governing cell cycle progression, hormonal homeostasis, angiogenesis, cell cycle progression, cell survival, and proliferation [15]. Moreover, accumulating evidence has shown that I3C has multiple therapeutic activities, including anti-ulcer [16], anti-adipogenic [17], anti-inflammatory [18, 19], anti-oxidant [20], cardioprotective [21], and cancer chemopreventive activities. Despite the fact that I3C possesses many potential therapeutic activities and advances in preclinical research, it has never been studied in experimental AD models. In the current study, we investigated the effect of I3C on AD pathogenesis using a 1-chloro-2,4-dinitrochlorobenzene (DNCB)-induced AD model and human keratinocytes.

## Materials and methods

### Chemicals and reagents

Sigma; EMD Millipore (Billerica, MA, USA) supplied I3C (I7256, ≥ 96%), 3-(4,5-dimethylthiazol-2-yl)−2,5-diphenyl tetrazolium bromide (MTT), dimethyl sulfoxide (DMSO), and all other chemicals for this study. Bio-Techne Ltd. (Abingdon, OX, UK) provided recombinant human tumor necrosis factor (TNF)-α and recombinant human IFN-γ. Life Technologies, Inc. (Grand Island, NY, USA) supplied Dulbecco’s modified Eagle’s medium (DMEM), fetal bovine serum (FBS), penicillin, and streptomycin. Primary antibodies against p-p38 (cat no. 9211), p38 (cat no. 9212), c-Jun N-terminal kinase (JNK) (cat no. 9252), p-JNK (cat no. 9251), and p-TAK1 (cat no. 4508) were acquired from Cell Signaling Technology Inc. (Danvers, MA, USA). Santa Cruz Biotechnology, Inc. (Dallas, TX, USA) provided primary antibodies against periostin (cat. sc-398631), TAK1 (cat. sc-7967), p-IκB-α (cat. sc-8404), IκB-α (cat. sc-203), TSLPR (cat. sc-293312), involucrin (cat. sc-21748), loricrin (cat. sc-9542), Ah receptor (cat. sc-133088), and β-actin (Cat. sc-81178). Abcam (Cambridge, UK) and Novus Biologicals (Centennial, CO, USA) were the sources of TSLP (ab188766) and loricrin, respectively. Jackson ImmunoResearch Laboratories Inc. (West Grove, PA, USA) supplied horseradish peroxidase-conjugated secondary antibodies. R&D Systems Inc. (Minneapolis, MN, USA) provided ELISA kits for TNF-α, IL-1β, IL-6, IgE, and TSLP. Takara Bio, Inc. (Shiga, Japan) supplied SYBR Premix Ex Taq, and Bioneer Corporation (Daejeon, South Korea) provided oligonucleotide primers.

### Cell viability and sample treatment

Provided by Professor Jae-Young Um (Kyung Hee University, Republic of Korea), HaCaT keratinocytes underwent cultivation at 37 °C in DMEM supplemented with 10% FBS, penicillin (100 U/mL), and streptomycin (100 μg/mL) within a humidified atmosphere of 5% CO_2_. To determine the toxicity of I3C on HaCaT keratinocytes, an MTT assay was performed. Cells were seeded in 96-well culture plates at a density of 5 × 10^4^ cells/mL in culture medium and allowed to attach for 24 h. Cells were treated with medium containing various concentrations of I3C. After incubation for 24 h, the cells were treated with 50 μL of MTT (5 μg/mL) for 4 h. For investigation purposes, cells were seeded at a density of 1 × 10^5^ cells per well, incubated for 24 h, and subjected to treatment with I3C at concentrations of 25, 50, and 100 μM for 1 h at 37 °C in a humidified environment with 5% CO_2_. Subsequently, the cells were stimulated with TNF-α (10 ng/mL) and IFN-γ (10 ng/mL) at 37 °C, and the formazan precipitate was dissolved in DMSO. Absorbance was measured at 540 nm using a microplate reader.

### DNCB-induced AD model

Acquired from Charles River Laboratories (Harlan Laboratories, Inc., Wilmington, MA, USA), thirty NC/Nga male mice (6 weeks old; 20–25 g body weight) were meticulously maintained under consistent conditions: a temperature range of 20–25 °C, humidity between 40–60%, and a 12-h light/dark cycle. Randomly assigned to one of the five groups (n = 6 per group), the mice underwent induction of AD-like symptoms and skin lesions using DNCB. For the induction of AD-like symptoms and skin lesions, DNCB was employed. In a concise overview, the dorsal skin of the mice underwent stripping with cellophane tape. Subsequently, it was topically sensitized with 100 μL of 1% DNCB dissolved in a 4:1 v/v mixture of acetone and olive oil, applied to the shaved area of the dorsal surface for three consecutive days, followed by a four-day period of no treatment. Following the initial challenge that induced AD-like symptoms, the treatment regimen involved repeated stripping and application of 100 μL of 0.5% DNCB for a duration of 28 days. Mice received topical administration of either vehicle (acetone/olive oil), dexamethasone (5 mg/kg admixed in Vaseline), or I3C (50 or 100 mg/kg oral administration) 4 h after each DNCB treatment, once a day over a span of 4 weeks. At the experiment's conclusion, lymph nodes and spleens were obtained for body weight comparison, and skin tissues were subjected to histological and western blot analyses. All procedures adhered to university guidelines and received approval from the Ethical Committee for Animal Care and the Use of Laboratory Animals, Korean Medicine, Sangji University (Wonju, Korea; approval no. 2021-05).

### Evaluation of dermatitis severity

The severity of clinical dermatitis, evaluated at the experiment's onset and conclusion, utilized the method outlined by Yamamoto and colleagues. The scoring system, encompassing the development of erythema/hemorrhage, scarring/dryness, edema, and excoriation/erosion, employed a scale of 0 for none, 1 for mild (< 20%), 2 for moderate (20–60%), and 3 for severe (> 60%). The cumulative sum of individual scores was employed as the dermatitis score.

### Cytokine and IgE assay

Culture media were collected post-treatment with I3C and stored at − 70 °C. The levels of TNF-α, IL-1β, and IL-6 were measured using ELISA kits, according to the manufacturer’s instructions. At the conclusion of the experiment, blood samples were procured from each mouse. Serum was acquired through centrifugation at 1700 × *g* for 30 min and stored at − 80 ℃ until analysis. IgE release was measured using an ELISA kit, according to the manufacturer’s protocol.

### Measurement of transepidermal water loss (TEWL)

The assessment of TEWL on the dorsal skin of NC/Nga mice was conducted at the 8-week mark using GPskin Barrier Light (Gpskin, Seoul, Republic of Korea), adhering to established protocols. TEWL in the mouse dorsal skin was measured under specific conditions at 24 °C and 50–55% humidity. Placing the probe at the center of the shaved dorsum area of each mouse recorded the TEWL value in g/m2/h. Statistical values were expressed as a fold change compared to the control group.

### Histopathological analysis

Dorsal skin samples were collected from the mice at the end of the study period. To detect epidermal thickness and inflammatory cells, the samples were fixed in 10% buffered formalin, embedded in paraffin, and sectioned into 4-μm-thick slices. Staining with hematoxylin and eosin (H&E) and toluidine blue was performed. Pathological changes in all stained skin sections were observed through a DM IL LED microscope (Leica, Wetzlar, Germany) and documented using a DFC295 camera (Leica, Wetzlar, Germany). Digital images were captured from each slide (three per group) and measured using the Leica Application Suite (Leica, Wetzlar, Germany).

For immunohistochemistry (IHC), skin tissue slides were deparaffinized in xylene, rehydrated at different concentrations of ethanol (100%, 95%, 90%, 80%, and 70%), and hydrated with water. To quench endogenous peroxidase activity, slides were incubated with 0.6% H_2_O_2_ in 50% MeOH. The slides were permeabilized with 0.3% Triton in PBS, pre-blocked with 10% NGS for 1 h, and then incubated overnight with a specific primary antibody at 4 °C. The sections were then washed three times and incubated for 1 h with HRP-labeled secondary antibodies at room temperature. The antibody–antigen interaction was visualized using chromogenic DAB with hematoxylin and eosin counterstaining. For immunofluorescence analysis, the skin sections were incubated with primary antibodies overnight at 4 °C. After washing, the slides were incubated with the secondary antibody Alexa Fluor 594-conjugated goat anti-mouse Invitrogen. Glass slides with mounted coverslips were utilized, and images were captured on a Leica TCS SP5 (LAS AF) microscope (Leica Microsystems) connected to a light microscope.

### Western blot analysis

For the suspension of segments from cells or dorsal tissues, PRO-PREP™ protein extraction solution (Intron Biotechnology, Inc., Seoul, Korea) was employed, followed by a 20-min incubation at 4 °C. Cell debris elimination involved microcentrifugation at 11,000 × *g* for 30 min at 4 °C, succeeded by the swift freezing of the supernatant. Protein concentration determination utilized Bio-Rad protein assay reagent (Bio-Rad Laboratories, Inc., Hercules, CA, USA) as per the manufacturer’s protocol. Cellular proteins from treated and untreated cell extracts (10–30 μL) underwent electroblotting onto a polyvinylidene fluoride membrane after separation with 8–12% SDS-PAGE. The membrane was subject to a 1-h incubation with blocking solution (5% skim milk) at room temperature, followed by an overnight incubation with primary antibodies (1: 1000) at 4 °C. Subsequent washing three times with Tween 20/Tris-buffered saline (T/TBS) preceded a 2-h incubation with a horseradish peroxidase-conjugated secondary antibody (1:2000) at room temperature. Final steps involved three washes with T/TBS and development using enhanced chemiluminescence (GE Healthcare Life Sciences, Chalfont, UK). Densitometric analysis was executed with the Bio-Rad Quantity One software version 4.3.0 (Bio-Rad Laboratories, Inc., Hercules, CA, USA).

### Statistical analysis

Data are expressed as the mean ± standard deviation of triplicate experiments. Statistically significant differences were compared using one-way analysis of variance and Dunnett’s post-hoc test. P < 0.05 was considered a statistically significant difference. Statistical analysis was performed using the SPSS statistical analysis software (version 19.0, IBM SPSS, Armonk, NY, USA).

## Results

### I3C inhibited the pro-inflammatory response and expression of TSLP and periostin in HaCaT keratinocytes

**Fig. 1:**
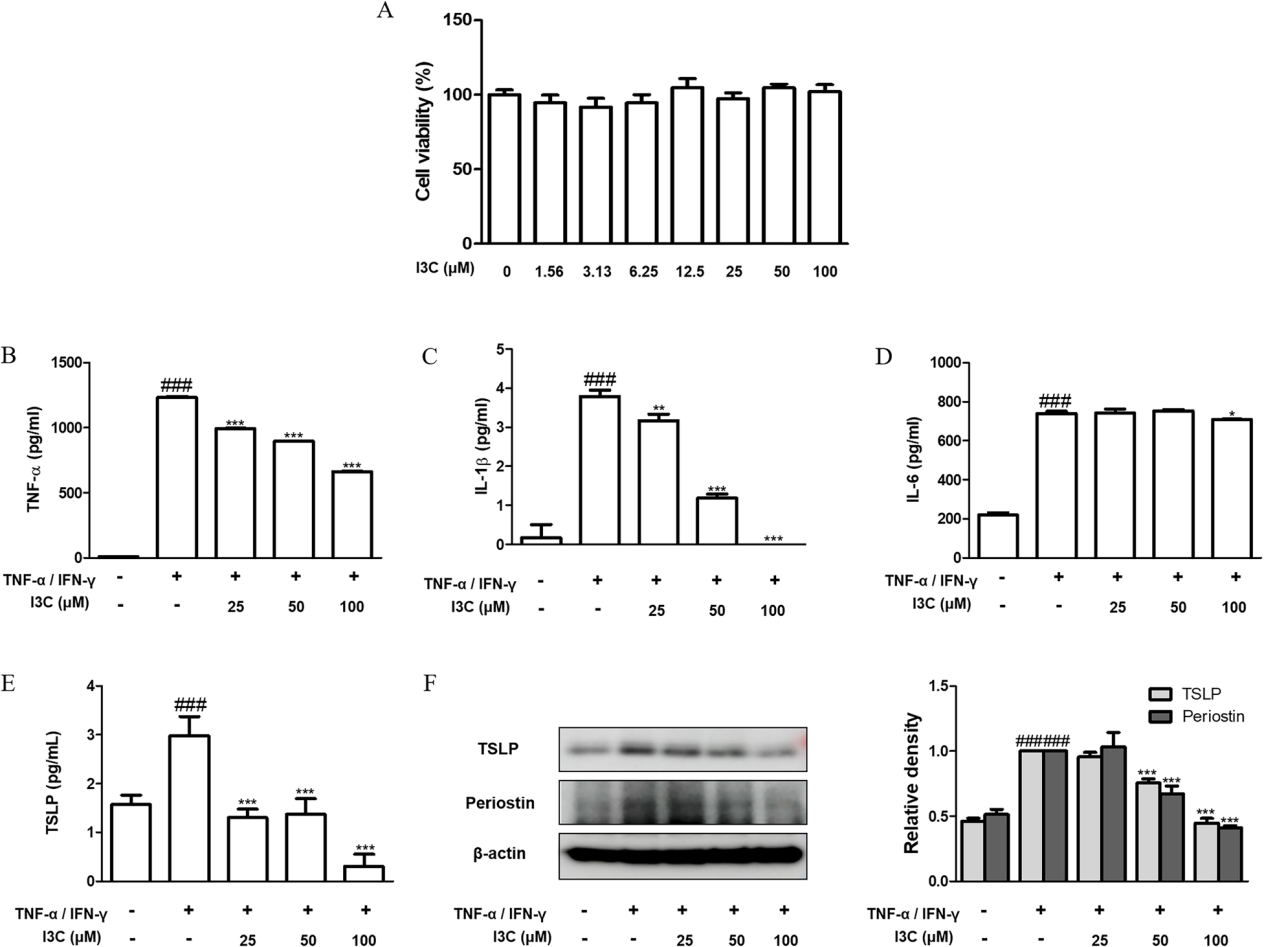
Effects of I3C on the pro-inflammatory cytokines, TSLP, and periostin in TNF-α/IFN-γ-stimulated HaCaT keratinocytes. **A** Cell viability was measured using MTT assay in HaCaT keratinocytes. **B–E** Pro-inflammatory cytokines productions were measured using ELISA kit. **F** TSLP and periostin were western-blotted from total proteins. Internal control featured β-actin. Densitometric analysis was facilitated using Bio-Rad Quantity One® Software. The data presented represent the mean ± S.D. of three independent experiments. ### p < 0.001 vs. the control group; *p < 0.05, and ***p < 0.001 vs. the TNF-α/IFN-γ-stimulated group

Conducting an MTT assay enabled the evaluation of I3C's cytotoxic impact on human HaCaT keratinocytes. Notably, I3C exhibited no cytotoxic effects at concentrations up to 100 μM (Fig. 1A), prompting further in vitro investigations at concentrations of 25, 50, and 100 μM. The study proceeded to analyze I3C's influence on the production of pro-inflammatory cytokines, namely TNF-α, IL-1β, IL-6, and TSLP, in keratinocytes stimulated with TNF-α and IFN-γ. Results indicated a significant elevation in cytokine secretion following stimulation compared to baseline conditions, yet pre-treatment with I3C led to a decrease in TNF-α, IL-1β, IL-6, and TSLP levels in HaCaT keratinocytes (Fig. 1B–E), with a more pronounced inhibitory effect observed on TNF-α and IL-1β. Given the recognized involvement of TSLP and periostin in AD pathogenesis [12], the study subsequently explored the impact of I3C on their expression. Western blot analysis revealed heightened TSLP and periostin expression in response to TNF-α and IFN-γ stimulation, which were attenuated upon treatment with I3C in HaCaT keratinocytes (Fig. 1F). These findings suggest a potential role for I3C in mitigating skin inflammation through the inhibition of TSLP and periostin expression.

### I3C alleviated DNCB-induced AD-like skin symptoms in mice

**Fig. 2:**
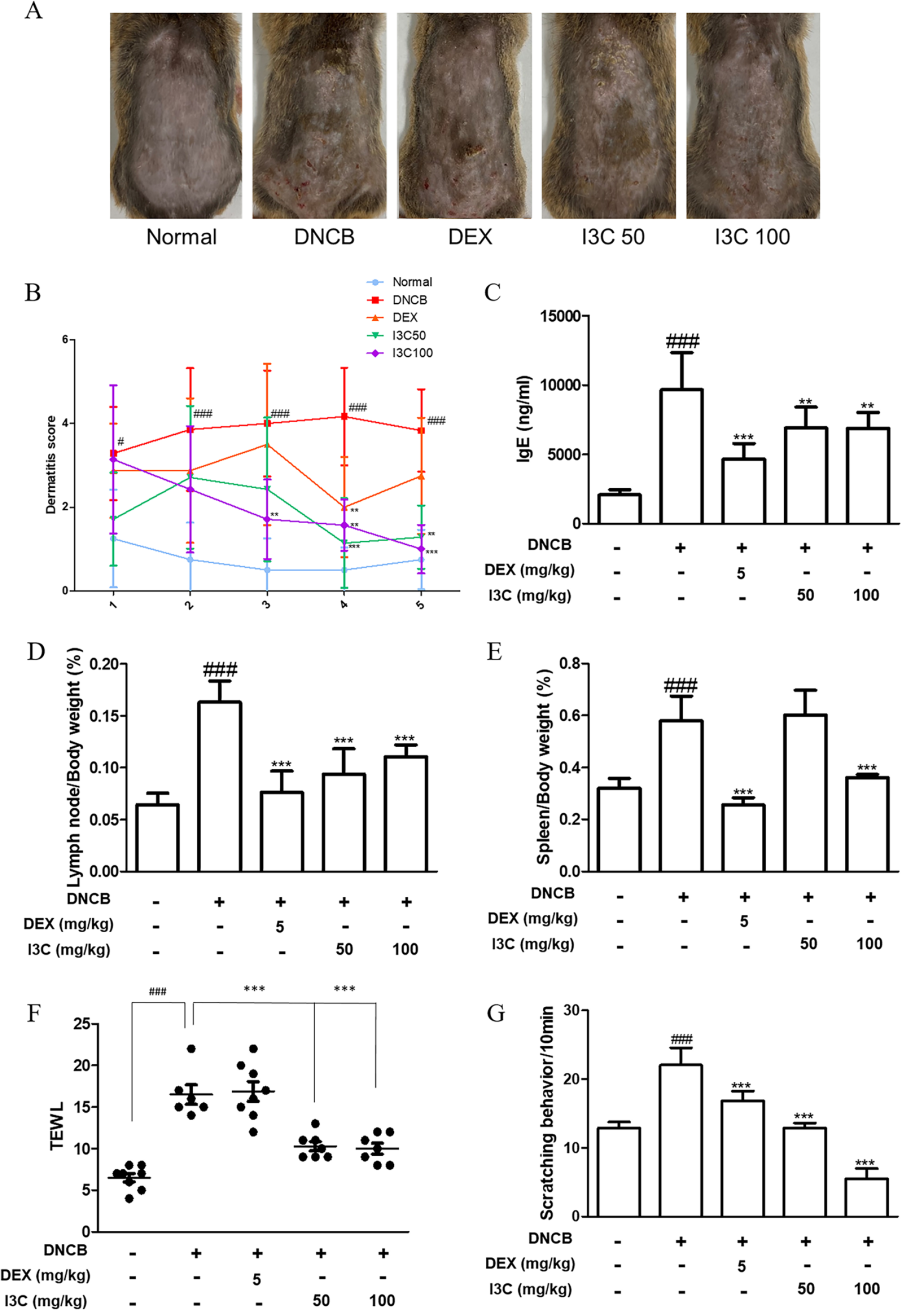
Effects of I3C on the clinical features of DNCB-induced AD skin in NC/Nga mice. **A** Clinical features of AD-skin symptoms of mice. **B** Measurement of dermatitis scores occurred once a week over 5 weeks. The dermatitis score, defined as the sum of scores graded for each symptom, was recorded. **C** Serum level of IgE was measured using an ELISA kit. **D** Lymph nodes and **E** spleen weights of mice were recorded and shown as weight/body weight ratio (%). **F** Transepidermal water loss (TEWL) and **G** scratching number of mice were measured at end of 5 weeks. ### p < 0.001 vs. the control group; *p < 0.05, **p < 0.01, and ***p < 0.001 vs. DNCB-stimulated group

**Fig. 3:**
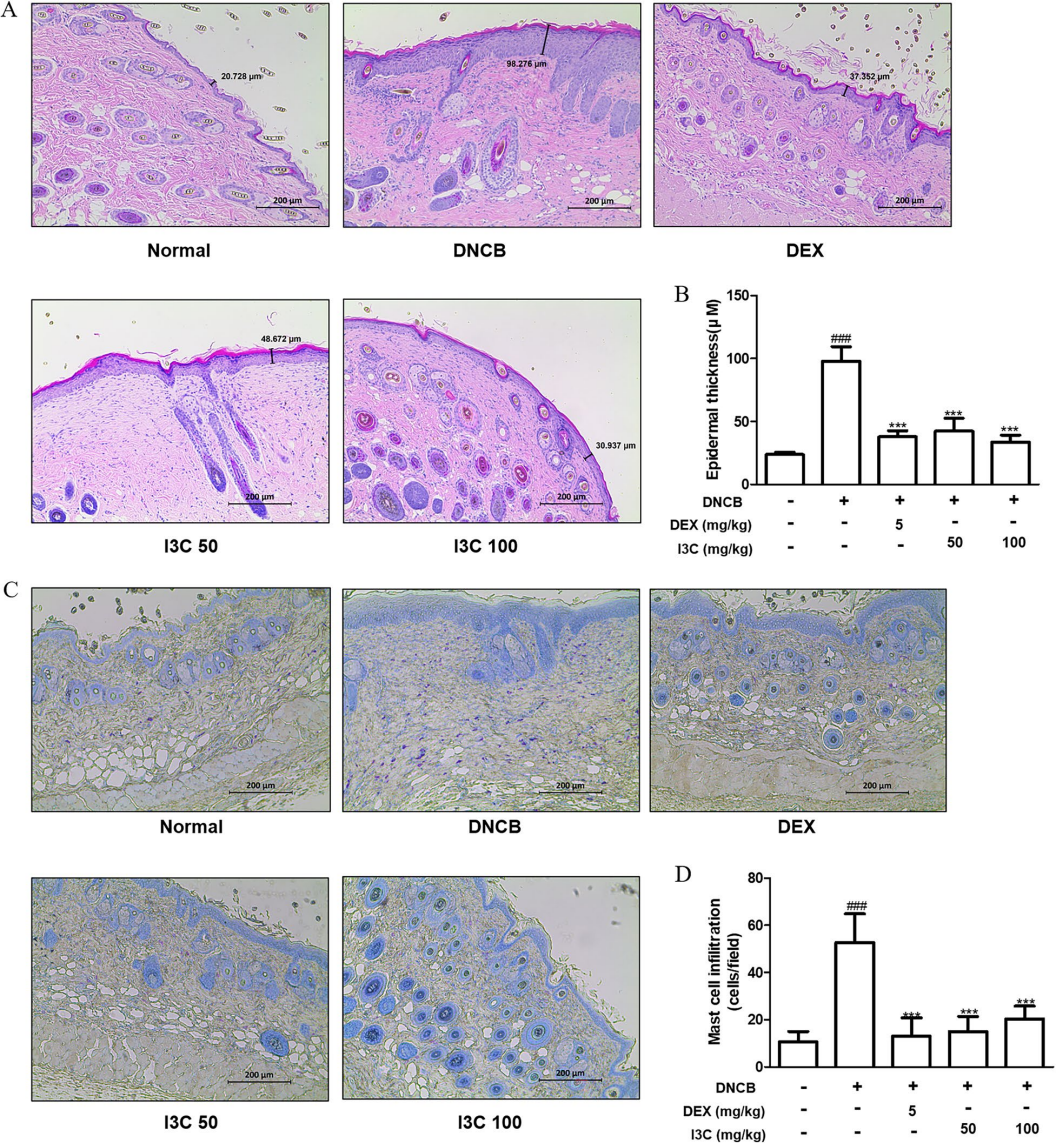
Effect of I3C on histological alterations in DNCB-induced AD skin in NC/Nga mice. **A** AD mouse skin lesions stained with H&E, showcasing a scale bar of 200 μm. **B** Measurement of epidermal thickness involved assessing H&E stained sections under a microscope. **C** Presentation of AD mouse skin lesions stained with toluidine blue, featuring a scale bar of 200 μm. **D** Mast cell infiltration in toluidine blue stained sections is quantified as the average total count across five fields. The presented data signify the mean ± S.D. of three independent experiments. ### p < 0.001 vs. the control group; ***p < 0.001 vs. the DNCB-stimulated group

For the purpose of validating the effects of I3C in an in vivo setting, an experimental model of AD induced by DNCB was devised. Sensitization of mouse dorsal skin was achieved through the application of 1% DNCB, subsequently leading to the development of AD-like skin inflammation. Evaluation of the results revealed pronounced clinical manifestations on the dorsal skin of DNCB-challenged mice, characterized by erythema, edema, scarring, dryness, excoriation, and hemorrhage (Fig. 2A), accompanied by elevated dermatitis scores and serum levels of IgE (Fig. 2B and C). Remarkably, oral administration of I3C significantly mitigated AD-like symptoms and reduced IgE serum levels in the mice (Fig. 2A–C). Furthermore, DNCB-induced enlargement of axillary lymph nodes and spleens was observed, which was notably reversed by I3C treatment, leading to decreased lymph node and spleen size and weight (Fig. 3D and E). Additionally, trans-epidermal water loss (TEWL) and the frequency of scratching behaviors in I3C-treated mice were significantly diminished compared to DNCB-treated mice, with levels even lower than those observed in the positive control DEX group (Fig. 3F and G).

### I3C attenuated histological alterations in skin of DNCB-induced AD mice

The histopathological assessment of dorsal skin tissue, conducted through H&E and toluidine blue staining, provided additional insights into the impact of I3C on the histological characteristics of AD in mice. DNCB-challenged mice exhibited notable epidermal thickening and infiltration of inflammatory cells (Fig. 3A and B). Additionally, toluidine blue staining revealed notable mast cell infiltration within the dermis of DNCB-induced AD mice (Fig. 3C and D). Nevertheless, oral administration of I3C significantly mitigated epidermal thickening and reduced the abundance of mast cells within the skin tissue of AD mice (Fig. 3A–D).

### I3C decreased the periostin and TSLP expression and recovered skin barrier proteins in the skin of DNCB-induced AD mice

**Fig. 4:**
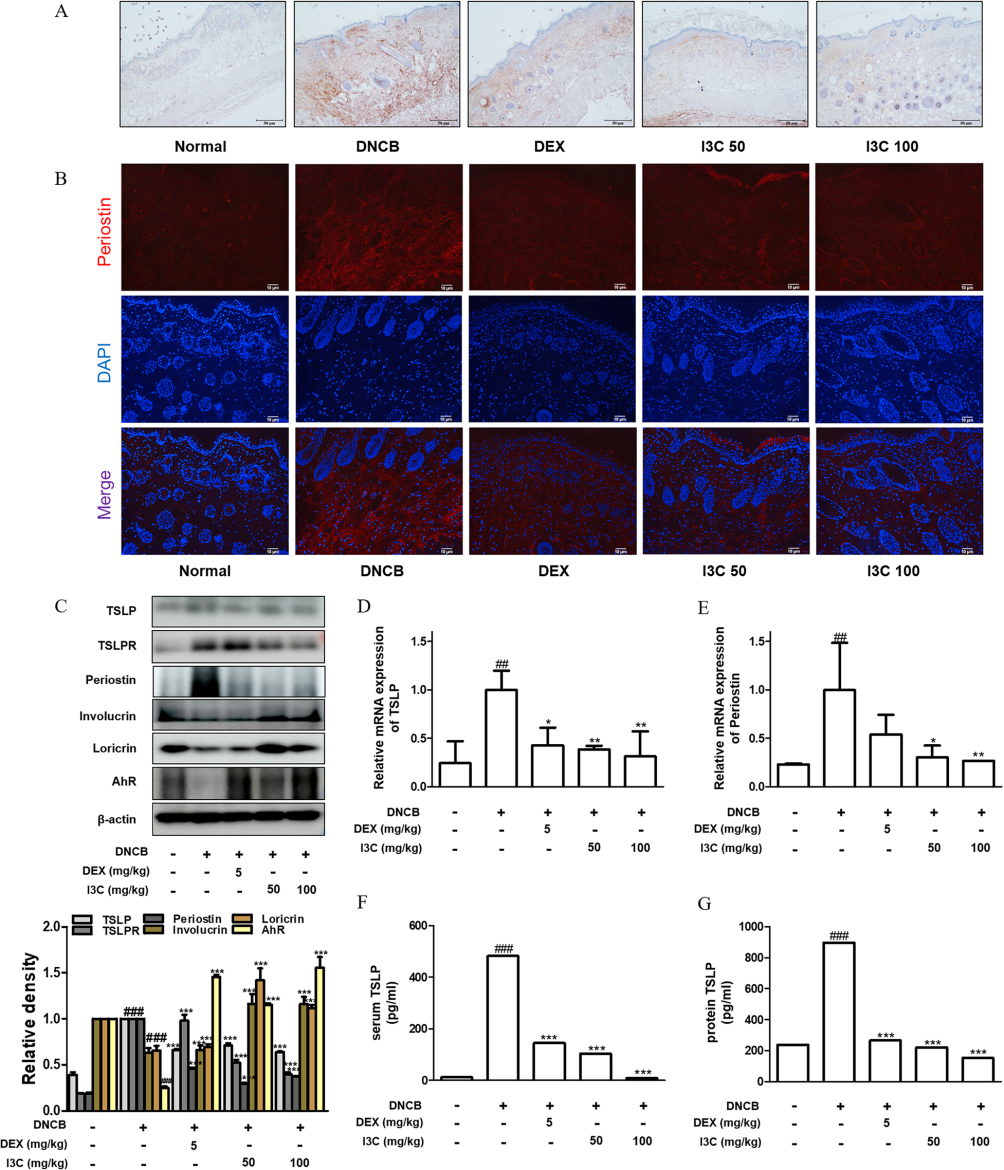
Effects of I3C on TSLP, periostin, and skin barrier proteins via AhR expression in DNCB-induced AD skin in NC/Nga mice. Periostin expressions in mouse dorsal skin. Skin sections stained by **A** immunohistochemistry (scale bar = 50 μm) or **B** immunofluorescence with anti-periostin antibody. Red: periostin staining, blue: DAPI nuclei staining (scale bar = 10 μm). **C** TSLP, TSLPR, periostin, involucrin, loricrin, and AhR were the focus of western blotting in total protein preparations. Specific antibodies facilitated this analysis, with β-actin serving as the internal control. Densitometric assessment was conducted using Bio-Rad Quantity One® Software. The mRNA expression of **D** TSLP and **E** periostin was determined using RT-qPCR based on GAPDH as the internal control. **F** Serum level of TSLP and **G** protein level of TSLP were measured using an ELISA kit. The data depicted convey the mean ± S.D. of three independent experiments. ### p < 0.001 vs. the control group; ***p < 0.001 vs. the DNCB-stimulated group

To explore the contribution of periostin to the pathogenesis of AD, we initially conducted a comparative analysis of periostin expression in skin lesions obtained from AD-afflicted mice. Utilizing immunohistochemical and immunofluorescent staining techniques, we observed a significant upregulation of periostin expression in the lesional skin of mice induced with DNCB-induced AD in contrast to skin samples from normal mice. Notably, periostin accumulation within the dermal region of the skin was particularly evident. However, upon treatment with I3C, there was a noticeable downregulation of periostin expression in the skin (Fig. 4A and B). Furthermore, as depicted in Fig. 4C, the substantial reduction in TSLP, TSLP receptor (TSLPR), and periostin expression was observed in the skin of mice administered with I3C compared to those in the DNCB group. Subsequently, we examined alterations in the expression levels of epidermal proteins, specifically involucrin and loricrin, crucial constituents involved in forming the skin barrier. In the skin of DNCB-induced AD mice, diminished levels of involucrin and loricrin were observed compared to the normal group, whereas a pronounced increase was evident in the skin of mice treated with I3C. Furthermore, the analysis of the Aryl Hydrocarbon Receptor (AhR) demonstrated that DNCB treatment decreased AhR expression, whereas I3C treatment increased AhR levels beyond control levels, suggesting that I3C acts as an AhR activator crucial for skin homeostasis and barrier function [22–24]. Besides, PCR analyses revealed that I3C treatment significantly reduces mRNA levels of TSLP and periostin (Fig. 4D and E), and this effect is corroborated by decreased TSLP levels in serum and protein samples of ELISA results (Fig. 4F and G). In conclusion, this research showed that I3C-induced activation of AhR regulates TSLP expression and TSLPR binding and then subsequently affects periostin expression, thereby contributing to reduced inflammatory responses and improved skin barrier function such as involucrin and loricrin.

### I3C regulated p38, JNK MAPKs and NF-κB pathway in skin of DNCB-induced AD mice

**Fig. 5:**
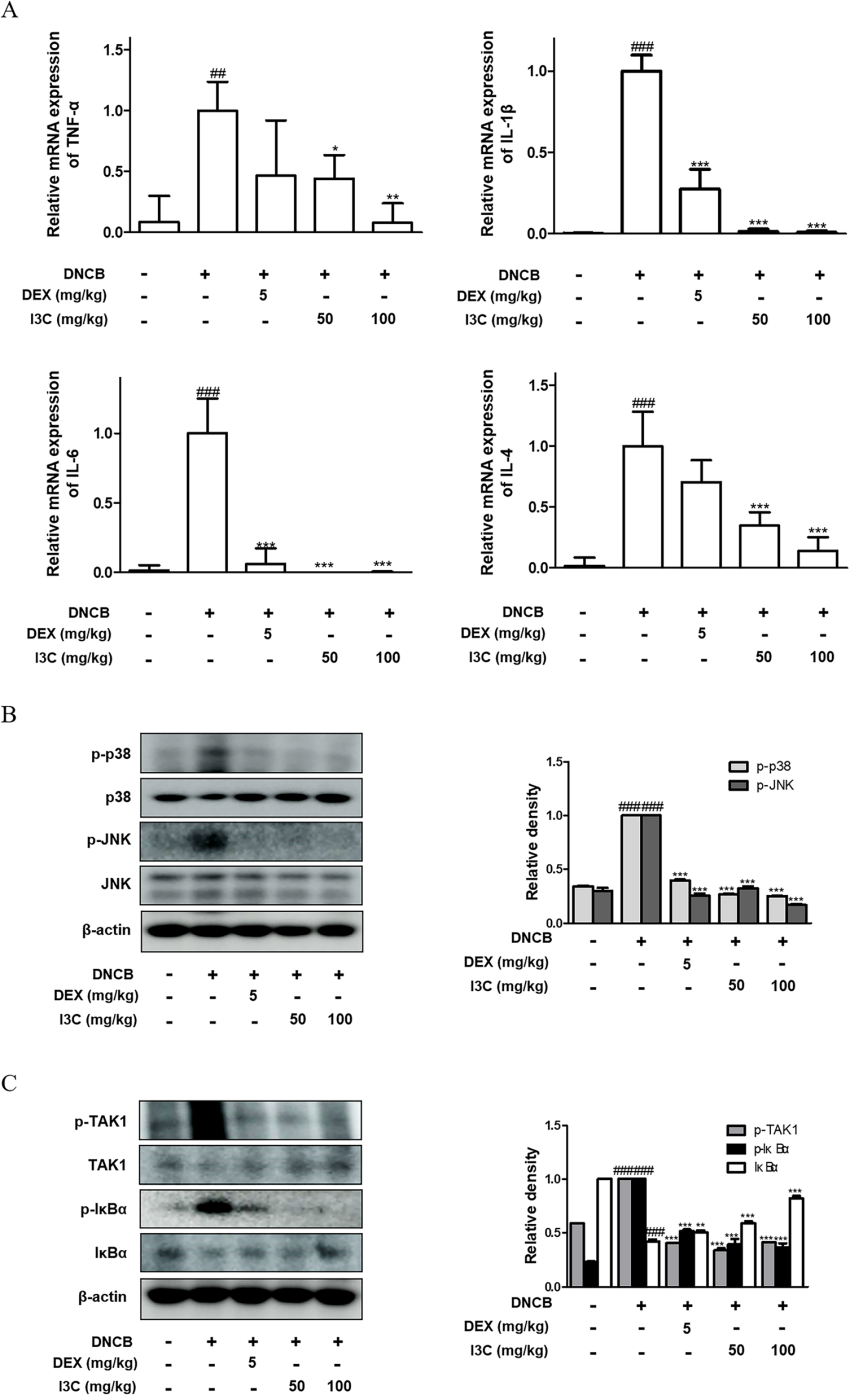
Effects of I3C on the activation of MAPKs and NF-κB pathway in DNCB-induced AD skin in NC/Nga mice. **A** The mRNA expression of TNF-α, IL-1β, IL-6 and IL-4 was determined using RT-qPCR based on GAPDH as the internal control. Total proteins were prepared and western blotted for **B** p-JNK, JNK, p-p38, p38, **C** p-TAK1, TAK1, p-IκBα, and IκBα using specific antibodies. Internal control β-actin guided the western blotting for this segment. Densitometric analysis, facilitated by Bio-Rad Quantity One® Software, was applied to the data, illustrating the mean ± S.D. of three independent experiments. ### p < 0.001 vs. the control group; **p < 0.01, and ***p < 0.001 vs. the DNCB-stimulated group

To demonstrate the effect of I3C on the cytokine levels in the dorsal tissue of DNCB-induced AD mice, the mRNA expression of TNF-α, IL-1β, IL-6 and IL-4 were measured. The result showed that a significant reduction in expression of TNF-α, IL-1β, IL-6 and IL-4 in I3C-treated group (Fig. 5A). To expand our understanding of the underlying mechanisms in vivo, we investigated the potential molecular pathways through which I3C exerts its inhibitory effects on AD-like skin inflammation in mice induced with DNCB-induced AD. Our focus shifted towards examining the impact of I3C on components of the mitogen-activated protein kinase (MAPK) and nuclear factor-κB (NF-κB) signaling pathways. As illustrated in Fig. 5B, DNCB treatment led to heightened phosphorylation levels of p38 and JNK MAPK compared to those observed in the normal group, whereas these levels markedly decreased in the skin of mice treated with I3C. Moreover, DNCB exposure resulted in increased phosphorylation levels of TAK1 and IκBα, critical regulators of NF-κB activation; however, treatment with I3C led to a notable reduction in phosphorylation levels in DNCB-induced AD mice (Fig. 5C).

## Discussion

AD poses a formidable challenge in the realm of healthcare, lacking effective preventive measures or definitive treatments. Its onset stems from a multifaceted interplay of environmental and genetic factors [25], with immune-inflammatory complexities often heralding the development of asthma and other allergic conditions [26]. Periostin has recently emerged as a promising biomarker of type 2 inflammation in allergic diseases [27], notably induced by signature type 2 cytokines like IL-4 and IL-13 or exacerbating their effects, thereby propagating allergic skin inflammation [28]. Moreover, periostin assumes a critical role in AD pathogenesis by orchestrating the release of pro-inflammatory cytokines and chemokines, such as TSLP, IL-25, and IL-33, from activated keratinocytes [11]. In light of this, our study sought to investigate the therapeutic potential of targeting periostin and TSLP in AD-like skin inflammation. To establish an AD-like skin inflammation model, we devised a mouse model involving sensitization and challenge with DNCB, a commonly employed skin irritant. Our findings revealed that repeated DNCB applications upregulated TSLP and periostin expression concurrent with scratching-induced responses, epidermal hyperplasia, and compromised skin barrier integrity. Notably, our observations align with previous reports demonstrating that recurrent sensitization with house dust mite induces periostin accumulation in the dermis of mice [11].

This investigation marks the inaugural study to propose the anti-inflammatory effects of I3C on skin keratinocytes and in the AD animal model. Our observations revealed that I3C markedly reduced the production of pro-inflammatory cytokines, notably TNF-α and IL-1β, emanating from activated keratinocytes (referenced in Fig. 1B–D). The stimulation of cytokines in keratinocytes and the application of DNCB on mouse dorsal skin significantly elevated the expression levels of TSLP and periostin, which were effectively diminished by I3C intervention (as depicted in Figs. 1E and 4). Notably, we detected elevated serum IgE levels in DNCB-treated mice, a response instigated by the Th2 cell-mediated activation of B cells [29]; however, these levels were substantially reduced following I3C treatment (illustrated in Fig. 2C). Th2 cytokines are known to suppress the expression of critical skin barrier proteins such as filaggrin, involucrin, and loricrin, leading to allergic skin inflammation and barrier impairment [30]. Our findings align with this understanding, showing an escalation in TSLP and periostin levels concomitant with the reduction of the skin barrier proteins involucrin and loricrin in DNCB-induced AD-like skin conditions, which were subsequently ameliorated by I3C treatment (as shown in Fig. 4C). Nonetheless, significant expression of filaggrin in skin lesions was not detected in our study, either in keratinocytes or in DNCB-treated mice (data not presented). Through this study, we have elucidated the anti-inflammatory properties of I3C on AD-like skin, highlighting its potential to inhibit TSLP and periostin expression and modulate Th2 responses and skin barrier functionality.

Periostin serves as a mediator of epithelial–mesenchymal transition via the MAPK signaling pathway [31] and it activates the NF-κB pathway through integrins, thereby contributing to the activation of epithelial/mesenchymal interactions within the skin [32]. Its significance as a matricellular protein in allergic diseases lies in its ability to bind to various integrin molecules on cell surfaces, thereby providing signals for tissue development and remodeling [33]. Accumulated periostin directly influences keratinocytes to produce cytokines such as TSLP by binding to αv integrin receptors on their surface. Notably, among these integrins, αvβ3 is recognized for its ability to activate NF-κB, a process critical for TSLP expression [34]. Intriguingly, several studies have investigated the suppressive effect of I3C on NF-κB activation [15, 35], underscoring the significance of NF-κB inhibition within keratinocytes in the context of allergic skin inflammation. Furthermore, our study suggests that I3C mitigates AD-related skin inflammation by downregulating TSLP/periostin expression and inhibiting the signaling pathways associated with TSLP/periostin, including NF-κB and MAPK (depicted in Fig. 5). The wide range of activities has been assigned to I3C, notably as an inhibitor of NF-κB [35] and MAPK [36]. Weng et al. demonstrated that [1-(4-chloro-3-nitrobenzenesulfonyl)−1H-indol-3-yl]-methanol (CIM), an I3C derivative, has potential as the antipsoriatic molecule usint a psoriasis-like mouse model where TNFα was employed as the stimulator to activate HaCaT cells [37]. It was found that CIM exhibits anti-inflammatory ability by downregulating MAPK signaling and NF-κB phosphorylation in keratinocytes, which is consistent with our study. Moreover, CIM was shown to reduce MAPK phosphorylation, thereby decreasing the expression of phospho-activator protein-1 (AP-1) (p–c-Jun and p–c-Fos) induced by TNF-α. Further investigation into detailed signaling pathways such as AP-1 is needed in the present study, alongside research involving various cells associated with the complex mechanisms of AD.

Furthermore, I3C demonstrates therapeutic potential by inhibiting the expression of TSLP and periostin, thus showcasing its anti-inflammatory and immunomodulatory properties. Through its ability to significantly reduce TSLP and periostin levels in HaCaT keratinocytes, as depicted in Fig. 1, I3C presents a promising approach for targeting inflammatory pathways implicated in AD. This inhibition holds particular relevance for AD treatment, as TSLP and periostin play crucial roles in initiating and sustaining allergic responses, contributing to symptoms such as pruritus, erythema, and skin barrier dysfunction [38, 39], as evidenced in Figs. 2 and 3. Moreover, the downregulation of periostin expression by I3C, as shown in Fig. 4, suggests its potential to mitigate tissue remodeling and chronic AD lesions. Interestingly, blocking the MAPK and NF-κB signaling pathways using inhibitors showed a reduction in cytokine-induced TSLP levels, and the effect of the inhibitors was further synergized when treated with I3C (Supplementary figure S1a). Considering the properties of I3C as an activator of AhR, which operates independently of the MAPK and NF-κB signaling pathways, we hypothesized that AhR would exert its effects. We confirmed that the reduced levels of AhR in DNCB-induced AD dorsal tissue were restored by I3C treatment (Fig. 4C). Given that AhR is a crucial regulator of skin barrier function, promoting the expression of barrier-related molecules, the synergistic effect of I3C on skin barrier function was further evidenced by the restoration of filaggrin mRNA levels in keratinocytes excepting the JNK inhibitor-treated group (Supplementary figure S1b). Thus, I3C can still activate the AhR pathway and other alternative pathways, potentially preserving its therapeutic benefits. Modulating the AhR and nuclear factor-erythroid 2-related factor 2 (NRF2) pathways is emerging as a novel approach in treating AD and other inflammatory skin diseases. Activation of these pathways by agonists has downstream effects on epidermal barrier function, immunomodulation, oxidative stress reduction, and cutaneous microbiome modulation [40]. AhR expression and signaling are altered in many inflammatory skin disorders such as AD and psoriasis, and clinical trials with tapinarof (first-in-class, non-steroidal, topical AhR agonist) have validated AhR as a therapeutic target capable of delivering significant efficacy [41]. Understanding the intricate interactions and compensatory mechanisms within cellular signaling can aid in developing therapeutic strategies that fully harness I3C's potential. Further research is required to explore these interactions and their clinical implications.

I3C is abundant in Cruciferae or Brassicaceae family plants, and medicinal herbs like *Raphanus sativus* Linné, *Brassica campestris* L., and *Isatis indigotica* Fort. are emblematic of this family in traditional Chinese medicine [42, 43]. Since I3C is primarily found in Cruciferae vegetables and has various therapeutic benefits [44, 45], there's a need for research on its content in Cruciferae herbs, including those mentioned. Furthermore, I3C is known for its rapid absorption and metabolism, resulting in quick distribution to various organs. According to the report of Mark et al. [46], mice were administered I3C at a dose of 250 mg/kg via gavage, and blood samples collected after 15 min showed plasma concentrations of 24.53 ± 5.48 μg/ml. In the present study, mice were given a dose of I3C at 100 mg/kg. For cell treatment, the high concentration used is 100 μM, which is approximately equivalent to 14.717 μg/ml. This is comparable to the in vivo concentration achieved in the mice, suggesting that the cells are exposed to a similar level of I3C. However, it is important to note that I3C is rapidly cleared from the bloodstream. Within 1 h post-administration, I3C becomes undetectable due to its metabolism into various metabolites, such as 3,3′-diindolylmethane (DIM), [2-(Indol-3-ylmethyl)-indol-3-yl]indol-3-ylmethane (LTr(1)), and 1-(3-hydroxymethyl)-indolyl-3-indolylmethane (HI-IM). The rapid clearance of I3C and the subsequent rise in its metabolites mean that the biological impact of I3C is not only due to the parent compound but also significantly influenced by its metabolites. Therefore, when interpreting the results of studies involving I3C, the contribution of these metabolites should be taken into account to fully understand the pharmacological effect of I3C. I3C, its metabolites, and derivatives have been confirmed to have safe dosages, with numerous clinical trials conducted to prevent and treat conditions such as obesity, chronic inflammation, lupus erythematosus, and various cancers [44]. The anti-inflammatory effects of I3C are anticipated to extend beyond skin conditions to encompass a broad spectrum of inflammatory diseases, including immune and metabolic disorders. Additional in-depth studies derived from this study could clarify the composition and therapeutic efficacy of these herbal medicines, providing valuable insights into their pharmacological properties.

## Conclusions

This study underscores the relevance of TSLP/periostin in AD-like skin and keratinocytes, hinting at the therapeutic potential of I3C in addressing TSLP/periostin-associated pathophysiology. However, elucidating the impact of I3C on skin-constituting cells beyond keratinocytes is imperative, necessitating further exploration of the molecular mechanisms underlying I3C's actions within the skin. Given the emerging roles of TSLP and periostin as diagnostic markers in conditions like asthma and obstructive lung disease, it is anticipated that I3C may exert multifaceted therapeutic effects against various diseases, including allergic skin inflammation. Our investigation reveals the collaborative involvement of the MAPK/NF-κB and AhR pathways in DNCB-induced AD-like skin inflammation and barrier dysfunction orchestrated by TSLP and periostin, effects substantially mitigated by I3C treatment. These findings underscore the therapeutic potential of targeting TSLP/periostin in AD skin pathogenesis and advocate for the utility of I3C in managing skin inflammation, particularly in AD.

## Supplementary Information

Suplementary Material 1.

## Data Availability

The data of this study are available from the corresponding author upon reasonable request.

## Abbreviations

AD: Atopic dermatitis
AhR: Aryl hydrocarbon receptor
I3C: Indole-3-carbinol (I3C)
DNCB: 2,4-Dinitrochlorobenzen
TEWL: Transepidermal water loss
TSLP: Thymic stromal lymphopoietin
MAPK: Mitogen-activated protein kinase
NF-κB: Nuclear factor-κB
