# Supplementary material for: Indole-3-carbinol alleviates allergic skin inflammation via periostin/thymic stromal lymphopoietin suppression in atopic dermatitis
Source: Chin Med. 2024 Dec 26;19:177. doi: 10.1186/s13020-024-01042-5 (PMC11670403; doi:10.1186/s13020-024-01042-5)
Supplement: Supplementary file 1 — Suplementary Material 1. [file 13020_2024_1042_MOESM1_ESM.docx]

**Supplementary Figure**


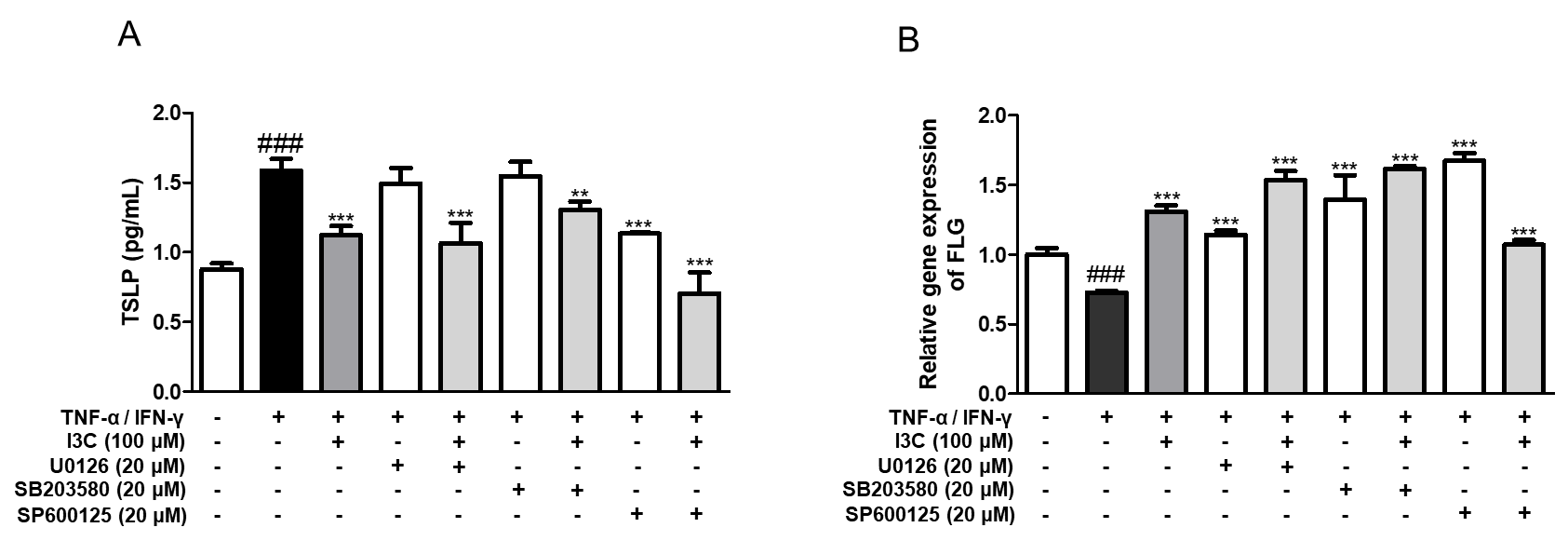


Figure S1. Effect of mitogen-activated protein kinase inhibition on the expression of TNF-α/IFN-γ-induced TSLP and FLG alteration. HaCaT cells were pretreated with U0126 (20 µM) as an ERK/ NF-κB inhibitor, SB203580 (20 µM) as a p38 inhibitor, or SP600125 (20 µM) as a JNK inhibitor, and with or without I3C (100 µM), followed by treatment with TNF-α/IFN-γ for 24 h. (A) Culture media were collected and stored at −70 °C. The levels of TSLP was measured using ELISA kits, according to the manufacturer’s instructions. (B) Total RNA was isolated, and the levels of FLG mRNA were measured using RT-PCR and quantitative real-time PCR. GAPDH mRNA was used as an internal control. The data presented represent the mean ± S.D. of three independent experiments. ^###^ p < 0.001 vs. the control group; ^*^p < 0.05, ^**^p < 0.01, and ^***^p < 0.001 vs. the TNF-α/IFN-γ-stimulated group.
